# Tracking of pedometer-determined physical activity in adults who relocate: results from RESIDE

**DOI:** 10.1186/1479-5868-5-39

**Published:** 2008-08-07

**Authors:** Catrine Tudor-Locke, Billie Giles-Corti, Matthew Knuiman, Gavin McCormack

**Affiliations:** 1 Walking Behavior Laboratory, Pennington Biomedical Research Center, Baton Rouge, LA, USA; 2 Centre for the Built Environment and Health, School of Population Health, The University of Western Australia, Crawley, WA, Australia; 3 Currently Population Health Intervention Research Centre, University of Calgary, Alberta, Canada, Formerly Centre for the Built Environment and Health, School of Population Health, The University of Western Australia, Crawley, WA, Australia

## Abstract

**Background:**

This secondary analysis investigated the extent and pattern of one-year tracking of pedometer-determined physical activity in people who relocated within the same metropolitan area (T1: baseline and T2: post-relocation). Specifically, data were derived from the RESIDential Environment Project (RESIDE), a natural experiment of people moving into new housing developments.

**Methods:**

1,175 participants (491 males, age = 42.6 ± 12.7 years, BMI = 27.2 ± 9.9 kg/m^2^; 684 females, age = 41.2 ± 11.3 years, BMI = 25.4 ± 5.2 kg/m^2^) wore a Yamax pedometer (SW-200-024) for seven days during the same season at both time points. Pearson's product-moment and Spearman's rank order correlations were used to evaluate the extent of tracking of mean steps/day. Age categories were set as youngest-29.9 (19 was the youngest in males, 20 in females), 30–39.9, 40–49.9, 50–59.9, and 60-oldest (78 was the oldest in males, 71 in females). Change in steps/day was also described categorically as: 1) stably inactive < 7,500 steps/day; 2) decreased activity (moved from ≥ 7,500 to < 7,500 steps/day between T1 and T2); 3) increased activity (moved from < 7,500 to ≥ 7,500 steps/day between T1 and T2); and, 4) stably active ≥ 7,500 steps/day at both time points. Stratified analyses were used to illuminate patterns by sex, age, and BMI-defined weight categories.

**Results:**

Overall, there was a small (non-significant) decrease in steps/day between T1 and T2 (mean ± SD is -81 ± 3,090 with 95%CI -259 to 97). With few exceptions (i.e., older women), both Pearson's and Spearman's correlations were moderate (r = 0.30–0.59) to moderately high (r = 0.60–0.70). The relative change/stability in steps/day (cut at 7,500 steps/day) was not significant across age groups in males (χ^2 ^= 17.35, p = .137) but was in females (χ^2 ^= 50.00, p < .0001). In both males and females the differences across BMI categories was significant (χ^2 ^= 22.28, p = .001 and χ^2 ^= 15.70, p = .015, respectively). For both sexes, those in the obese category were more stably inactive (and less stably active) between assessment points compared with those who were categorized as normal weight.

**Conclusion:**

Despite relocation, Western Australian adults held their rank position to a moderate to moderately high extent over one year. Categorized and expressed as relative stability/change over time, sex, age, and BMI patterns were evident.

## Introduction

Tracking, with regards to physical activity behaviors, refers to the extent to which an individual maintains relative position or rank over time [1]. Patterns of tracking include those categorized by sex, age, and indicators of body mass index (BMI)-defined weight status and physical activity levels that meet suggested cut points for health-related benefits. To date, much of the tracking literature has focused on young populations as they transition through adolescence [2,3] and beyond to adulthood [4,5]. Tracking with objective monitoring instruments has only been conducted in small samples of very young children (using accelerometers) [6,7] and adolescents (using pedometers) [2]. Limited information is available about tracking within adult populations [8] or across simple life span events such as relocation of primary residence. More than 39 million Americans (or about 14% of the population) changed addresses in 2004–2005 [9]. In Australia, over 3 million people (or almost 18% of the population) moved residences in 2000–2001 [10].

To our knowledge, no previous attempt has been made to describe tracking of adult physical activity behavior (objectively monitored or otherwise) disrupted by relocation. Therefore, the purpose of this secondary analysis was to investigate the extent and pattern of tracking of pedometer-determined physical activity between measures taken one year a part in a sample that moved residences within Perth, Western Australia, in the interim.

## Methods

The RESIDential Environments Project (RESIDE) is a natural experiment to evaluate the impact of urban design policies intended to encourage more active transport behaviors through the creation of safe, convenient pedestrian-friendly neighborhoods with access to shops, transit and parkland. As part of its longitudinal design, people moving into housing developments in Perth, Western Australia, were invited to participate in recurrent surveys and physical activity assessment using pedometers. Details about the selection of housing developments and subsequent recruitment of participants are provided in another publication [11]. All participants received written information about the study and provided written consent before providing any personal data. The present manuscript focuses on participants' baseline (T1) and 12-month follow-up (T2; after having moved to their new home) data points and presents a novel analysis not previously published. Specifically, participants were sent a T1 questionnaire and pedometer up to three months before the anticipated completion of their new home. The T2 questionnaire was sent approximately 12 months later with the aim that questionnaires and pedometer monitoring be completed in the same season.

Participants were asked to wear a Yamax-Digiwalker pedometer (SW-200-024) for seven days. This model has been shown to provide valid and reliable data [12,13]. Participants were asked to attach the pedometer to the waist-band of their clothing or belt at the front of the hip. Day-end steps were recorded in a log each night before retiring to bed. Participants were instructed to not reset the pedometer, except at the start of the first day of wearing the device. Thus a cumulative step-count was captured. In addition, participants recorded whether the pedometer was worn each day (i.e., all day, some of the day or not at all) or if the device had been removed for any reason throughout the day (e.g., for bathing, showering, or swimming).

Pedometer-determined steps for each day (except for day 1) were derived from the cumulative count by subtracting the previous day's steps from the current day's steps. Data were then screened for extreme values. Data for any single day indicating < 1,000 steps were removed and values > 30,000 steps on any single day were truncated (i.e., replaced with 30,000 steps). Equivalent cut points have been used to identify outliers among younger individuals [14]. Moreover, these cut points appear reasonable for our data given that the minimum and maximum daily steps found in a previous population-based study of Western Australian adults was 982 and 20,221 steps, respectively [15]. Mean steps/day for T1 and T2 were computed from the total weekly steps divided by the total days (6.5 ± 1.3 days overall) the pedometer was worn.

Other variables extracted from the original data set included sex, age and BMI (based on self-reported height and weight) at T1. Overall, 1,813 participants completed questionnaires at T1, and 75% of these participants (n = 1,356) returned completed T2 questionnaires. Data were reduced to include only those cases with complete pedometer data at both time points, BMI at T1, and those women who were not pregnant at T2 and reported no children less than 2 years of age at T2 (these latter two to conservatively exclude women considered pregnant at either time point). In total, 51 cases were missing some pedometer data, 48 were missing BMI data, 25 women were pregnant at T2 and 57 women had children less than 2 years at T2. After reductions, the final analysis data set used herein comprised of 1,175 cases (491 males, age = 42.6 ± 12.7 years, BMI = 27.2 ± 9.9 kg/m^2^; 684 females, age = 41.2 ± 11.3 years, BMI = 25.4 ± 5.2 kg/m^2^). There were no significant differences in steps/day or BMI from the original full data set; however, those in the reduced data set were on average 6.9 years older (p < 0.000). Age categories were set as youngest-29.9 (19 was the youngest in males, 20 in females), 30–39.9, 40–49.9, 50–59.9, and 60-oldest (78 was the oldest in males, 71 in females).

Descriptive analysis included computation of mean ± SD pedometer-determined physical activity (steps/day) at T1, T2, and mean ± SD change (Δ) between T2 and T1. A negative Δ indicates a decrease over time; a positive Δ indicates an increase. Pearson's product-moment and Spearman's rank order correlations were used to evaluate extent of tracking of steps/day. Malina's [1] suggestions for interpreting correlations were used: < 0.30 is low, 0.30–0.59 is moderate, and ≤ 0.60 is moderately high. Patterns of tracking were explored across sex-and age-specific categories, and initial BMI-defined weight categories (normal weight = BMI < 25 kg/m^2^, overweight = BMI ≥ 25 kg/m^2 ^to < 30 kg/m^2^, and obese = BMI > 30 kg/m^2^) using ANOVA (first by sex and age category, and secondly by sex and BMI-defined weight category) and stratified analyses.

Change in steps/day was also described categorically as: 1) stably inactive < 7,500 steps/day; 2) decreased activity (moved from ≥ 7,500 to < 7,500 steps/day between T1 and T2); 3) increased activity (moved from < 7,500 to ≥ 7,500 steps/day between T1 and T2); and, 4) stably active ≥ 7,500 steps/day at both time points. The dichotomous cut point 7,500 steps/day was selected since evidence indicates that health benefits can be realized and that accepted public health recommendations are achievable at this threshold [16-18]. Stratified analyses were used to illuminate patterns by sex, age, and BMI-defined weight categories. Chi-square analyses were interpreted for significance. SPSS 15 was used and alpha was set at p < 0.05.

## Results

Table 1 presents pedometer-determined physical activity (steps/day) at T1, T2, and change between T2 and T1. ANOVA (with between-subjects factors of sex and age category) indicated an overall small but not quite significant change in steps/day (i.e., lower mean steps/day at T2; F = 3.724, df = 1, p = .054) and no significant effects (or interaction effects) of sex or age category. Overall, mean ± SD for Δ was -81 ± 3,090 steps/day with 95%CI (-259, 97).

**Table 1:** Sex-and age-category strata of mean ± SD steps/day

	**T1** ** Steps/day**	**T2 ** **Steps/day**	**Pearson's** ***r***	**Spearman's** ***r***	**Δ = T2 – T1** **Steps/day ** ** (95% CI)**
**Youngest-29.9 years**					

Males (n = 77)	9,610 ± 4,558	9,471 ± 4,247	.650*	.651*	-139 ± 3,695 (-977, 700)
Females (n = 103)	8,076 ± 3,324	7,981 ± 3,322	.567*	.566*	-94 ± 3,091 (-698, 510)

**30–39.9 years**					

Males (n = 148)	8,973 ± 3,430	8,932 ± 3,209	.627*	.541*	-41 ± 2,876 (-509, 427)
Females (n = 245)	8,859 ± 3,177	9,174 ± 3,091	.555*	.523*	315 ± 2,957 (-57, 687)

**40–49.9 years**					

Males (n = 118)	9,067 ± 3,720	8,660 ± 3,186	.610*	.560*	-407 ± 3,088 (-970, 156)
Females (n = 165)	9,017 ± 2,936	9,170 ± 3,080	.530*	.543*	153 ± 2,920 (-296, 602)

**50–59.9 years**					

Males (n = 89)	7,833 ± 2,908	7,733 ± 3,410	.529*	.533*	-100 ± 3,097 (-752, 553)
Females (n = 111)	8,749 ± 3,702	8,342 ± 3,189	.606*	.645*	-407 ± 3,091 (-988, 174)

**60-oldest**					

Males (n = 59)	7,982 ± 3,261	7,206 ± 3,298	.596*	.542*	-776 ± 2,946 (-1,544, -8)
Females (n = 60)	7,139 ± 2,780	6,732 ± 3,260	.238	.304*	-408 ± 3,747 (-1,376, 561)

**Total sample**					

Males (n = 491)	8,769 ± 3,635	8,527 ± 3,494	.621*	.572*	-243 ± 3,112 (-519, 33)
Females (n = 684)	8,610 ± 3,240	8,644 ± 3,235	.550*	.551*	34 ± 3,071 (-197, 264)

Note: 19 was the youngest in males, 20 in females; 78 was the oldest in males, 71 in females.*correlation is significant at the 0.01 level (2-tailed)

Table 1 also displays Pearson and Spearman correlations computed between T1 and T2 steps/day, collated by sex and age category. Overall, both Pearson and Spearman correlations were moderate to moderately high. This suggests that individuals within the groups held their rank position to a moderate extent between assessments. A singular exception was observed in females 60+ years of age; the Pearson correlation (0.238; low) was non-significant, however, the Spearman correlation (0.304; moderate) was statistically significant.

Table 2 presents sex-and BMI-defined weight category strata of pedometer-determined physical activity (steps/day) at T1, T2, change (Δ) between T2 and T1, and Pearson and Spearman correlations. ANOVA (with between-subjects factors of sex and BMI-defined category) revealed a significant effect of BMI-category on Δ steps/day (F = 3.100, df = 2, p = .045); no other effects (or interactions) were significant. Again, both Pearson and Spearman correlations were moderate to moderately high. This suggests that individuals within lower BMI-defined weight categories (i.e., normal weight) were relatively more stable in their rank position over time than those in higher BMI-defined weight categories (i.e., obese).

**Table 2:** Sex-and BMI-defined weight category strata of mean ± SD steps/day

	**T1** ** Steps/day**	**T2** ** Steps/day**	**Pearson's** ***r***	**Spearman's** ***r***	**Δ = T2 – T1** **Steps/day** ** (95% CI)**
**Normal weight = BMI < 25 kg/m^2^**					

Males (n = 167)	9,220 ± 3,808	9,066 ± 3,571	.691*	.669*	-157 ± 2,907 (-601, 287)
Females (n = 373)	8,870 ± 3,189	8,739 ± 3,178	.597*	.564*	131 ± 2,859 (-422, 160)

**Overweight ≥ BMI = 25 kg/m^2 ^to < 30 kg/m^2^**					

Males (n = 243)	8,944 ± 3,611	8,481 ± 3,542	.573*	.511*	-463 ± 3,306 (-880, -45)
Females (n = 205)	8,679 ± 3392	8,704 ± 3,308	.524*	.539*	25 ± 3,270 (-425, 475)

**Obese = BMI > 30 kg/m^2^**					

Males (n = 81)	7,318 ± 2,957	7,555 ± 2,961	.525*	.511*	238 ± 2,886 (-400, 876)
Females (n = 106)	7,564 ± 2,926	8,193 ± 3,284	.425*	.467*	629 ± 3,344 (-15, 1274)

*correlation is significant at the 0.01 level (2-tailed)

**Figure 1:**
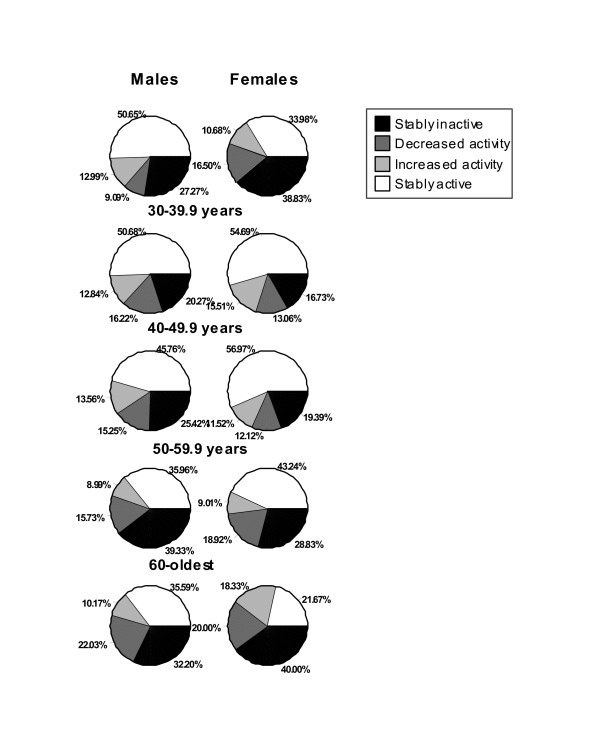
**Change/stability in steps/day (cut at 7,500 steps/day) by sex and age category**. Stably inactive < 7,500 steps/day; decreased activity (moved from ≥ 7,500 to < 7,500 steps/day between T1 and T2); increased activity (moved from < 7,500 to ≥ 7,500 steps/day between T1 and T2); and, stably active ≥ 7,500 steps/day at both time points.

The relative change/stability in steps/day (cut at 7,500 steps/day) by sex and age category is presented in Figure 1. In males, the difference in relative change/stability in steps/day across age categories was not significant (χ^2 ^= 17.35, p = .137), however, in females the difference was significant at (χ^2 ^= 50.00, p < .0001). Specifically, the proportion of females categorized as stably active was lower in the youngest age group, was higher in the second and third age groups, and lower again in the older age groups. Differences in the proportion of females who are stably inactive are a mirror image of this observation.

**Figure 2:**
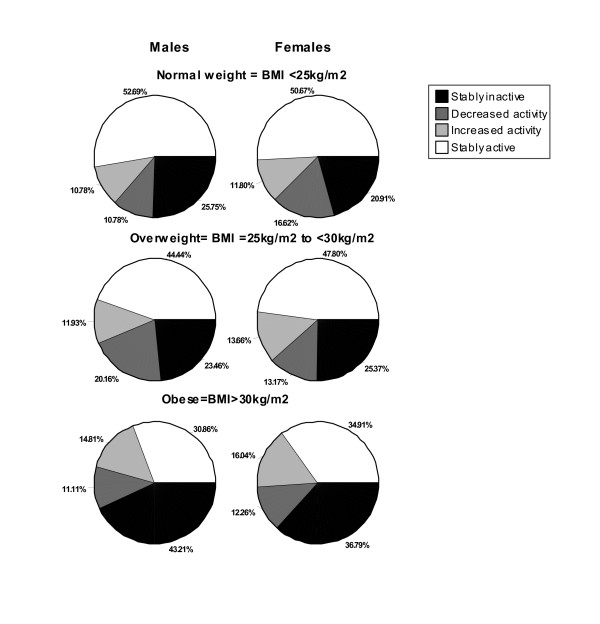
**Change/stability in steps/day (cut at 7,500 steps/day) by sex and BMI-defined weight category**. Stably inactive < 7,500 steps/day; decreased activity (moved from ≥ 7,500 to < 7,500 steps/day between T1 and T2); increased activity (moved from < 7,500 to ≥ 7,500 steps/day between T1 and T2); and, stably active ≥ 7,500 steps/day at both time points.

The relative change/stability in steps/day (cut at 7,500 steps/day) by sex and BMI-defined weight category is presented in Figure 2. In both males and females the differences across BMI categories was significant (χ^2 ^= 22.28, p = .001 and χ^2 ^= 15.70, p = .015, respectively). Notably, for both sexes, those in the obese category were more stably inactive (and less stably active) between assessment points than those who were categorized as normal weight.

## Discussion

These data represent the first known longitudinal pedometer data in adults. Previously, Raustorp and colleagues [2] reported pedometer tracking data for 97 Swedish adolescents assessed three times over five years, essentially capturing their development between 12 and 17 years of age. Pearson's correlations indicated low to moderate tracking in these adolescents, with patterns of higher tracking in boys than girls (i.e., correlations ranged from 0.55 in boys to 0.22 in girls). Most of the earlier tracking studies (children, adolescents, and adult) have relied on self-reported behaviors [1]. Spearman correlations for adult men and women were 0.31 and 0.23, respectively, in a large British cohort assessed on self-reported frequency of leisure physical activity at 33 and 42 years of age [8]. Spearman correlations ranged from -0.10 to 0.33 for self-reported time in physical activity assessed 7 years apart in a Canadian adult population [19]. In the present study, we found that pedometer-determined physical activity behavior tracks to a relatively higher extent over one year in Australian adults disrupted by relocation, than both pedometer-assessed physical activity in adolescents and self-reports of physical activity in adults. Specifically, most of the correlations evaluated herein (both Pearson's and Spearman's) fit within Malina's [1] suggested ranges for moderate to moderately high tracking of physical activity behavior. The primary exception was in older females. This group was comparatively less stable in behavior over time, that is, characterized by a less predictable shifting in rank order between time points. Although this group was relatively smaller in sample size, it was also somewhat lower in mean steps/day in addition to representing a more vulnerable age group.

A higher correlation is anticipated with a briefer time span between assessments [1]. Accordingly, the relatively higher correlations observed herein compared to the more prolonged duration between pedometer-based assessments of the Swedish adolescents [2] is somewhat anticipated. In contrast, however, Jackson et al. [6] reported correlations of 0.40 (Spearman) to 0.49 (Pearson) (again, generally lower than those we found) in 3–4 years old children assessed one year apart with accelerometers. Together, these findings suggest that physical activity behavior tracking may be more stable from year to year in adulthood, at least until older age groups (this last especially for women). Developing children and transitioning adolescents are exposed to continually changing personal, social, and physical environments, so some degree of instability in behavioral tracking is to be expected. The relative stability of these adults in their behavior, however, is especially interesting, given that the entire sample studied relocated between assessments.

The reasons that people relocate often differ as a function of their place in the lifecycle: younger and middle aged adults relocate for professional and personal opportunities while older adults often relocate for improved access to amenities and to be closer to family [20]. The process of relocation in generally considered a stressful life event [21], especially if it requires long distance moves accompanied with inevitable adjustments to new environments, services, and supports [20]. However, all of the participants in this study were already residents of Perth, Western Australia prior to relocating again within this community to different neighborhoods and locales; disruptions associated with long distance moves should therefore be considered minimal. Regardless the magnitude and scope of this now common life interruption, however, it is remarkable to note the overall stability of behavior (whether active or inactive) between time points in these adults. Overall, 25.9% of participants were stably inactive and 46.4% were stably active. From a health perspective, the higher proportion of those who are stably active is desirable, of course [2].

Exploring relative stability/change using a simple stratified analysis and Chi-square testing, we observed significant differential tracking patterns by sex, age, and BMI-defined weight categories. For example, when steps/day were categorized dichotomously and then evaluated for stability of such categorization over time, an age-related pattern was apparent for females, but not for males. However, it is also interesting to scrutinize the groups that changed their physical activity behavior over just one year. For example, of the ten specific sex-and-age strata studied, eight demonstrated a greater proportion of individuals who decreased their behavior compared to those who increased their behavior. This is an interesting finding worth pursuing in terms of confirmation and further exploration within other data sets; promoting maintenance of higher physical activity levels may be a separate and important approach to population health as opposed to focusing primarily on interventions directed at increasing behavior of sedentary individuals.

Overall, however, a tracking pattern of steps/day (expressed as a continuous variable) appeared to be most influenced by BMI-defined weight status. Further, relative stability of behavior (expressed as a categorical variable) appeared to be moderated by BMI-defined weight status for both sexes. To emphasize, normal weight individuals were more stable in their behavior (i.e., they maintained their position within the group) over time in contrast to those classified as obese. Scrutinizing Figure 2 further reveals that the proportion of obese individuals who increased their physical activity over the previous year was higher than those who decreased their behavior. Whether this phenomenon is merely regression to the mean (since those with higher BMI tend to take fewer steps/day) or a result of a wider spread personal decision to change behavior (perhaps driven by a motivation to influence weight) is a question worthy of future research.

A number of study limitations must be noted. Although this study of objectively monitored physical activity represents an improvement over self-report estimates, pedometers are not designed to detect intensity of movement. We are therefore unable to make firm conclusions about individual's participation in health-related quantities of at least moderate intensity physical activity, an outcome of public health interest [22]. However, using the cut point of 7,500 steps/day is a defensible proxy threshold value for a healthful level of physical activity [16-18]. Further, pedometers "miss" or underestimate non-ambulatory activities like weight training and cycling, and because they are not worn during water activities, swimming is not detected at all [23]. However, such activities account for only a small proportion of physical activity on a population level and the estimated average underestimation is approximately 300–700 steps/day [23]. Another limitation of this study is that weight and height were self-reported; estimates of overweight and obesity may therefore be underestimated [24]. Regardless of this potential bias, however, we were still able to observe clear moderating effects of BMI-defined weight categories on tracking of pedometer-determined physical activity. As is often the case for longitudinal studies, attrition is a threat to validity. Although study recidivism is evident, the data herein is reasonably representative of the originally recruited sample. These data, are of course, limited in their generalizability to populations most similar to Perth, Western Australia. Using similar pedometer brands, this sample (≅ 8,600 steps/day) was more similarly active compared to a previous independent Perth sample (≅ 9,500 steps/day) [15] than at least two U.S. samples: Colorado [25] (≅ 6,800 steps/day) and South Carolina (≅ 5,900 steps/day) [26].

In summary, in terms of pedometer-assessed physical activity, Western Australian adults held their rank position within groups to a moderate to moderately high extent (with few exceptions) between assessments separated by one year and disrupted by relocation. The observed pattern of change in steps/day was not influenced by sex or age, but was influenced by BMI-defined weight categories. Categorized and expressed as relative stability/change over time (i.e., stably inactive, decreased activity, increased activity, stably active), sex, age, and BMI patterns were evident. Of concern was the observation that individuals more frequently decreased (than increased) their physical activity over one year. Although not within the scope of this current study, it is possible to examine the additional demographic, personal, and environmental correlates of those categorized according to their relative stability or change between assessments points in future work.

## Competing interests

The authors declare that they have no competing interests.

## Authors' contributions

CTL conceived of the secondary analysis and lead analysis, interpretation and writing. BGC designed the original study, participated in design, interpretation and writing of this secondary analysis. MK contributed to the design of the original study and assisted with analysis, interpretation and writing. GMcC was involved in original data management and contributed to interpretation and writing. All authors read and approved the final manuscript.
